# Pterostilbene Inhibits Human Multiple Myeloma Cells via ERK1/2 and JNK Pathway In Vitro and In Vivo

**DOI:** 10.3390/ijms17111927

**Published:** 2016-11-17

**Authors:** Bingqian Xie, Zhijian Xu, Liangning Hu, Gege Chen, Rong Wei, Guang Yang, Bo Li, Gaomei Chang, Xi Sun, Huiqun Wu, Yong Zhang, Bojie Dai, Yi Tao, Jumei Shi, Weiliang Zhu

**Affiliations:** 1 Department of Hematology, Shanghai Tenth People’s Hospital, Tongji University School of Medicine, Shanghai 200072, China; xiebingqian0131@163.com (B.X.); inggiel@163.com (L.H.); 18767221930@163.com (Ge.C.); wei_rong001@163.com (R.W.); naiveyangboy@163.com (G.Y.); 13162975523@163.com (Ga.C.); 15026573070@163.com (X.S.); whq19691208@163.com (H.W.); 2 CAS Key Laboratory of Receptor Research, Drug Discovery and Design Center, Shanghai Institute of Materia Medica, Chinese Academy of Sciences, Shanghai 201203, China; zjxu@simm.ac.cn (Z.X.); boli@simm.ac.cn (B.L.); zhangyong109@simm.ac.cn (Y.Z.); 3 College of Life Science and Technology, Tongji University, Shanghai 200092, China; bojiedai@gmail.com

**Keywords:** multiple myeloma, pterostilbene, apoptosis, cell cycle, ERK1/2, JNK

## Abstract

Multiple myeloma (MM) is the second most common malignancy in the hematologic system, which is characterized by accumulation of plasma cells in bone marrow. Pterostilbene (PTE) is a natural dimethylated analog of resveratrol, which has anti-oxidant, anti-inflammatory and anti-tumor properties. In the present study, we examined the anti-tumor effect of PTE on MM cell lines both in vitro and in vivo using the cell counting kit (CCK)-8, apoptosis assays, cell cycle analysis, reactive oxygen species (ROS) generation, JC-1 mitochondrial membrane potential assay, Western blotting and tumor xenograft models. The results demonstrated that PTE induces apoptosis in the H929 cell line and causes cell cycle arrest at G0/G1 phase by enhancing ROS generation and reducing mitochondrial membrane potential. The anti-tumor effect of PTE may be caused by the activation of the extracellular regulated protein kinases (ERK) 1/2 and c-Jun N-terminal kinase (JNK) signaling pathways. Additionally, mice treated with PTE by intraperitoneal injection demonstrated reduced tumor volume. Taken together, the results of this study indicate that the anti-tumor effect of PTE on MM cells may provide a new therapeutic option for MM patients.

## 1. Introduction

Multiple myeloma (MM) is the second most common malignancy in the hematologic system after non-Hodgkin lymphoma, and it is characterized by the accumulation of plasma cells in bone marrow and secretion of monoclonal immunoglobulins [1,2]. MM is also associated with osteoclast activation and osteoblast suppression that can lead to lytic bone lesions, as well as the damage of end-organs, could induce anemia, immunodeficiency and renal dysfunction [3]. Although the existing therapies such as bortezomib, thalidomide and lenalidomide can enhance remission rates and prolong survival of MM patients, MM is still regarded as incurable due to the common development of drug resistance and therapeutic complications associated with the disease [4]. Therefore, it is necessary to establish novel effective therapies to treat MM. 

Resveratrol is a natural compound found in grapes, blueberries and cranberries, which has been reported to have anti-tumor properties due to its ability to induce apoptosis and cell cycle arrest [5]. Previous work has confirmed that resveratrol can inhibit the proliferation of human MM cell lines and alter the population of S phase [6]. Moreover, resveratrol can enhance the apoptotic effect of bortezomib and thalidomide, thus overcoming the chemoresistance of MM cells [2]. 

Pterostilbene (*trans*-3,5-dimethoxy-4′-hydroxystilbene, PTE) is a natural dimethylated analog of resveratrol found mainly in blueberries. PTE has greater bioavailability, a greater half-life and lower toxicity compared to resveratrol due to the lipophilicity of the two-methoxyl groups [7,8]. PTE has been reported to have anti-oxidant, anti-inflammatory, cardiovascular protective and anti-tumor effects, by enhancing apoptosis and inhibiting angiogenesis and metastasis [9]. The anti-tumor effect of PTE has been described in many types of cancers, including lung [10], breast [11] and prostate cancer [12]. Moreover, in the hematopoietic system, PTE has been reported to elicit an anti-tumor response in leukemia cell lines in vitro, and was associated with the induction of apoptosis and cell cycle arrest [13,14]. As PTE is an analog of resveratrol, which has known anti-tumor effects on MM cells, PTE may provide a novel chemical therapy for the patients with MM.

In the present study, we demonstrate the anti-tumor effect of PTE on MM cell lines both in vitro and in vivo. We reveal that PTE can induce apoptosis and cause cell cycle arrest in MM cell lines in vitro and inhibit tumor growth in vivo in a mouse xenograft model. Altogether, the results of this study indicate that PTE may be a promising candidate to treat MM.

## 2. Results

### 2.1. Pterostilbene (PTE) Inhibits the Proliferation of Multiple Myeloma (MM) Cell Lines

**Figure 1:**
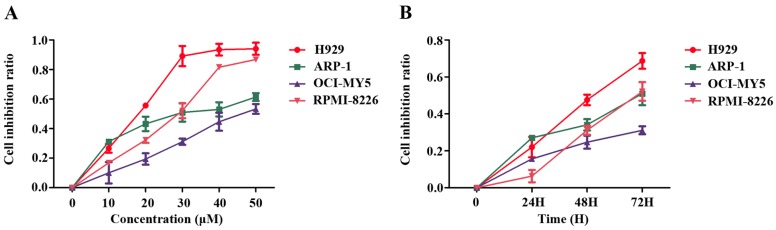
Pterostilbene (PTE) inhibits the proliferation of Multiple myeloma (MM) cell lines. (**A**) MM cells (H929, ARP-1, OCI-MY5 and RPMI-8226) were treated with PTE (10, 20, 30, 40 and 50 μM) in 96-well plates for 48 h; and (**B**) MM cell lines were treated with PTE (30 μM) in 96-well plates for 24, 48 and 72 h. Cell counting kit (CCK)-8 was used to monitor the cell proliferation.

To investigate whether PTE can inhibit the proliferation, the MM cell lines H929, ARP-1, OCI-MY5 and RPMI-8226 were treated with increasing doses of PTE (10, 20, 30, 40 and 50 μM) for 24, 48 and 72 h. Administration of PTE resulted in a dose-dependent decrease in MM cell proliferation, with the half maximal inhibitory concentrations (IC_50_) of H929, ARP-1, OCI-MY5 and RPMI-8226 at 72 h 15.37 ± 0.98, 26.15 ± 3.6, 43.46 ± 4.46 and 23.58 ± 0.41 μM, respectively (Figure 1A). Moreover, the anti-proliferative effect of PTE on MM cell lines appeared to occur in a time-dependent manner (Figure 1B).

### 2.2. PTE Enhances Caspase Activation and Induces Apoptosis in H929 Cells

**Figure 2:**
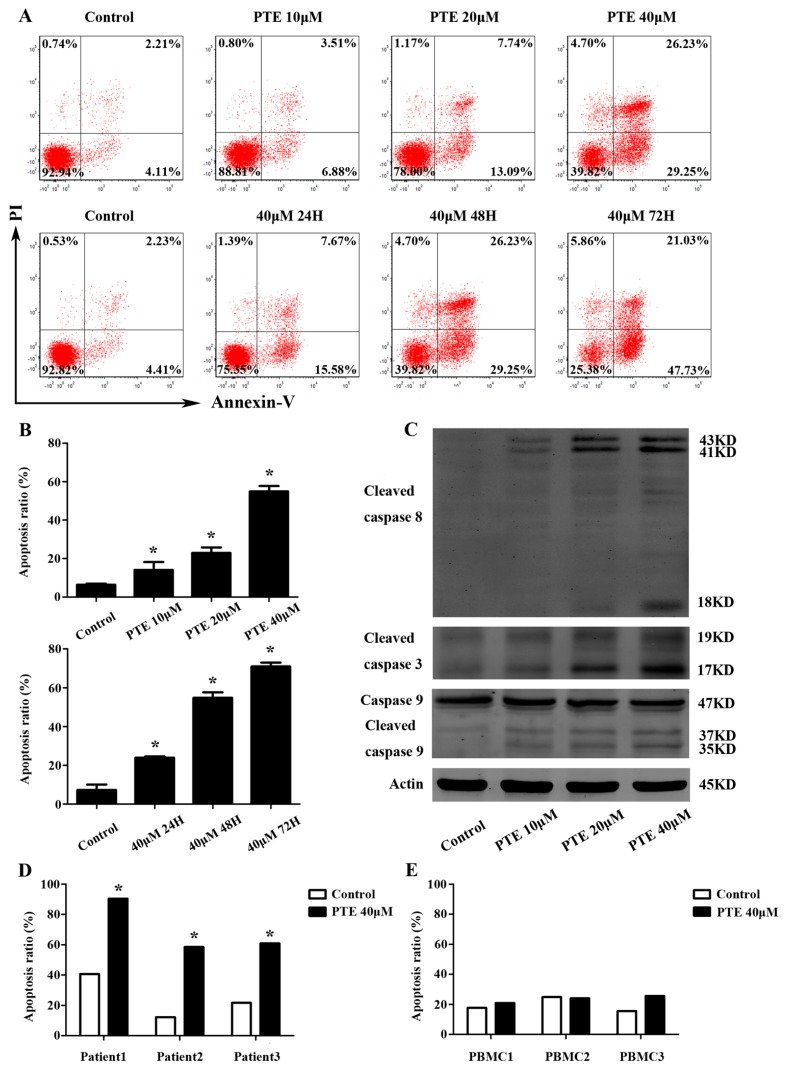

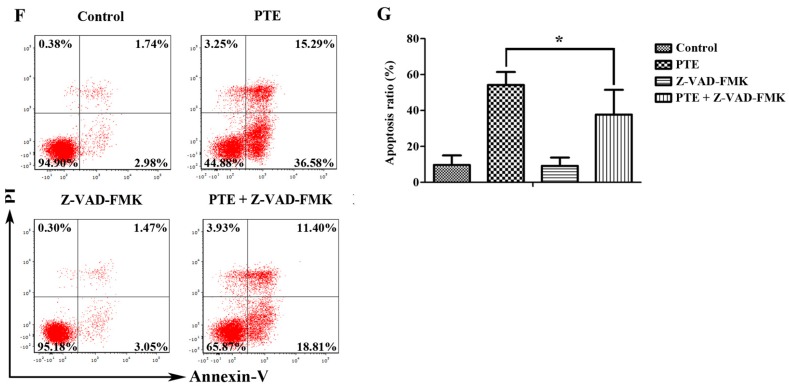
PTE enhances the apoptosis of H929 cells, primary CD138^+^ MM cells and caspase activation. (**A**) H929 cells were treated with PTE (10, 20, and 40 μM) for 48 h or treated with 40 μM for 24, 48 and 72 h, stained with AnnexinV- fluorescein isothiocyanate (FITC) / propidium iodide (PI) and analyzed by flow cytometry; (**B**) the percentage of FITC positive cells treated with 0, 10, 20, and 40 μM of PTE. Data is presented as mean ± SD (*n* = 3, * *p* < 0.05); (**C**) the protein levels of cleaved caspase-3, cleaved capase-8 and caspase-9 were determined by Western blot; (**D**,**E**) primary CD138^+^ MM cells and peripheral blood mononuclear cells (PBMCs) were treated with 40 μM of PTE for 48 h stained with AnnexinV-FITC/PI and analyzed by flow cytometry; (**F**) H929 cells were pre-incubated with or without Z-VAD-FMK (50 μM) for 3 h and then treated with PTE (40 μM) for 48 h, stained with AnnexinV-FITC/PI and analyzed by flow cytometry; and (**G**) the percentage of FITC positive cells treated with 40 μM of PTE that pre-incubated with or without Z-VAD-FMK. Data is presented as mean ± SD (*n* = 3, * *p* < 0.05).

Following PTE treatment, apoptosis was induced in H929 cells in a time- and dose-dependent manner, consistent with our findings in the CCK-8 assay (Figure 2A). Compared to the control group (6.36% ± 0.61%), apoptosis was increased at 48 h in the 10 μM (14.09% ± 0.61%), 20 μM (22.91% ± 2.84%) and 40 μM (54.87% ± 2.85%) PTE-treated group. Interestingly, we observed increased apoptosis at 24 h (23.95% ± 0.71%), 48 h (54.87% ± 2.85%) and 72 h (71.02% ± 1.95%) in the group treated with 40 μM PTE compared with controls (7.33% ± 2.76%) (Figure 2B). To further investigate the molecular mechanism by which PTE induces apoptosis in H929 cells, we assessed the activation of cleaved caspase-3, cleaved caspase-8, and caspase-9. As demonstrated in Figure 2C, PTE treatment resulted in increased levels of cleaved caspase-3, cleaved caspase-8 and cleaved caspase-9, indicating that PTE may induce both the extrinsic and intrinsic apoptotic pathway. Furthermore, a pan-caspase inhibitor can block the apoptosis of H929 cells treated with PTE (Figure 2F,G). Additionally, PTE was able to induce apoptosis in primary CD138^+^ MM cells without any obvious effects on normal peripheral blood mononuclear cells (PBMCs) (Figure 2D,E), suggesting that PTE may be a safe and non-toxic agent for treatment of MM.

### 2.3. PTE Arrests the Cell Cycle at G0/G1 Phase

**Figure 3:**
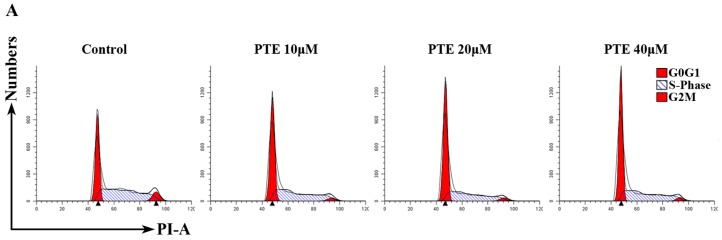

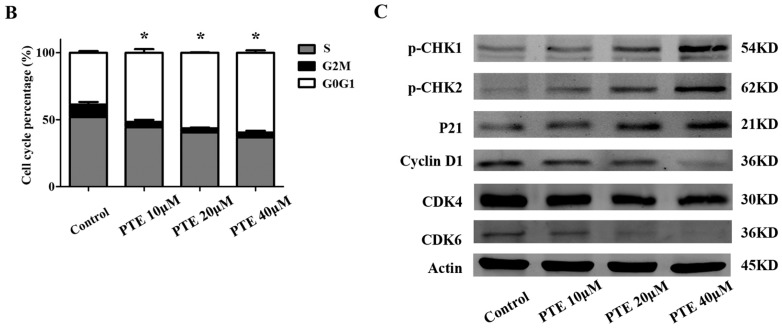
PTE arrests the cell cycle at G0/G1 phase. (**A**) H929 cells were treated with PTE (10, 20, and 40 μM) for 6 h, stained with PI and analyzed by flow cytometry; (**B**) the percentage of G0/G1, G2M and S phase following control or PTE treatment (10, 20, and 40 μM). Data is reported as mean ± SD (*n* = 3, * *p* < 0.05); and (**C**) the protein levels of phosphorylated checkpoint kinase (p-CHK) 1, p-CHK2, cyclin-D1, cyclin-dependent kinases (CDK) 6, CDK4 and p21 as assessed by Western blot.

As both apoptosis and the cell cycle are important for cell proliferation, we assessed the cell cycle following PTE treatment in H929 cells. The percentage of G0/G1 phase cells was increased with PTE treatment at 10 μM (51.62% ± 2.6%), 20 μM (56.42% ± 0.26%) and 40 μM (59.52% ± 1.66%) compared to the control group (38.75% ± 1.14%) (Figure 3A,B) at 6 h, indicating that PTE can arrest H929 cells at the G0/G1 phase. To further investigate the molecular mechanisms of PTE induced cell cycle arrest, we detected the activation of phosphorylated checkpoint kinase (p-CHK) 1, p-CHK2, cyclin-dependent kinases (CDK) 4, CDK6, cyclin-D1 and p21. PTE increased the activation of p-CHK1, p-CHK2 and p21, but decreased the levels of CDK4, CDK6 and cyclin-D1.

### 2.4. PTE Causes DNA Damage and Enhances Reactive Oxygen Species (ROS) Generation

**Figure 4:**
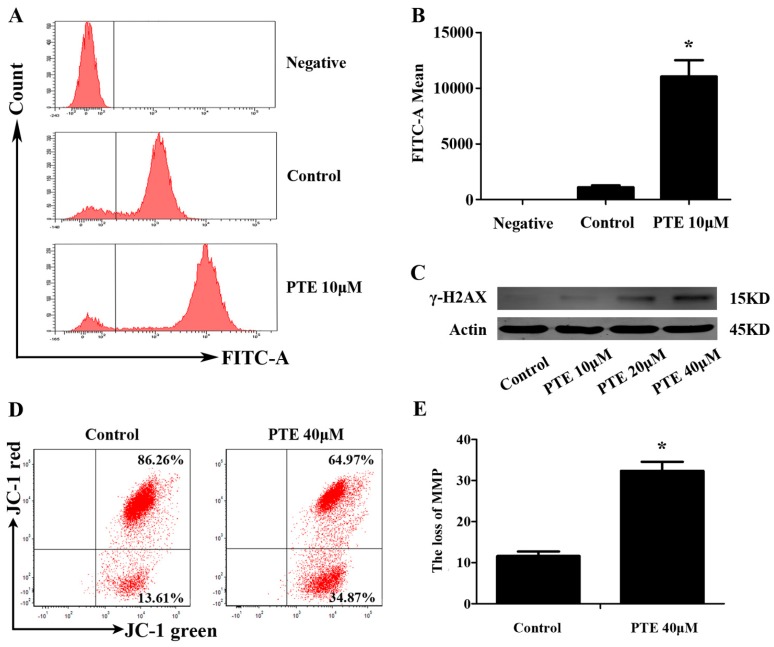

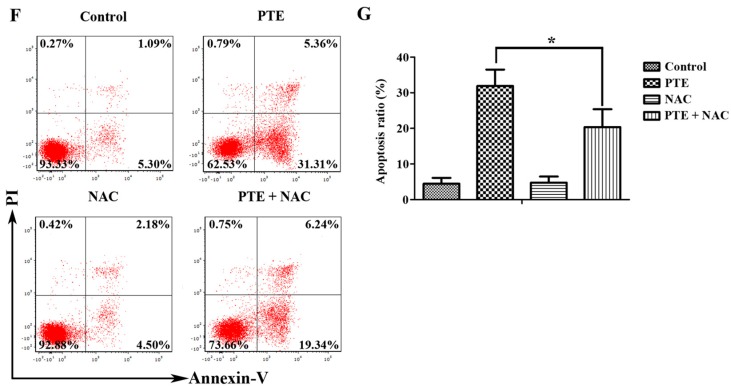
PTE enhances the level of reactive oxygen species (ROS) generation and loss of mitochondrial membrane potential (MMP). (**A**) H929 cells were treated with PTE (10 μM) for 24 h, and the level of ROS was detected by flow cytometry; (**B**) the fluorescence intensity of negative, control and PTE treatment groups. Data is presented as mean ± SD (*n* = 3, * *p* < 0.05); (**C**) the protein levels of γ-H2AX as assessed by Western blot; (**D**) MM cells were treated with PTE (40 μM) for 24 h and MMP was analyzed by flow cytometry; (**E**) JC-1 mitochondrial membrane potential assay green positive cells in control and PTE treatment groups. Data is presented as mean ± SD (* *p* < 0.05, *n* = 3); (**F**) H929 cells were pre-incubated with or without *N*-acetyl-l-cysteine (NAC) (10 mM) for 2 h and then treated with PTE (20 μM) for 48 h, stained with AnnexinV-FITC/PI and analyzed by flow cytometry; and (**G**) the percentage of FITC positive cells treated with 20 μM of PTE that pre-incubated with or without NAC. Data is presented as mean ± SD (*n* = 3, * *p* < 0.05).

Previous studies have reported that PTE can enhance the level of reactive oxygen species (ROS) generation in solid tumors. Consequently, we investigated ROS generation following PTE treatment in H929 cells by flow cytometry. ROS generation in the PTE treatment group (11047.67 ± 1454.12) was significantly increased compared to the control group (1109.33 ± 156.72) (Figure 4A). Furthermore, a ROS inhibitor can block the apoptosis of H929 cell lines treated with PTE. (Figure 4F,G) Additionally, we detected increased expression of γ-H2AX in response to PTE treatment, indicating that PTE induces DNA damage in H929 cells (Figure 4C).

### 2.5. PTE Induces the Loss of Mitochondrial Membrane Potential

We previously suggested that PTE might induce apoptosis via both the extrinsic and intrinsic apoptotic pathway. In order to further confirm the relationship between PTE and the intrinsic apoptosis pathway, we analyzed mitochondrial membrane potential by JC-1 mitochondrial membrane potential assay. Indeed, PTE treatment resulted in loss of mitochondrial membrane potential (32.34% ± 2.22%) compared with the control group (11.61% ± 1.11%) (Figure 4D).

### 2.6. PTE Enhances the Activation of Extracellular Regulated Protein Kinases (ERK) 1/2 and c-Jun N-Terminal Kinase (JNK) Pathway

**Figure 5:**
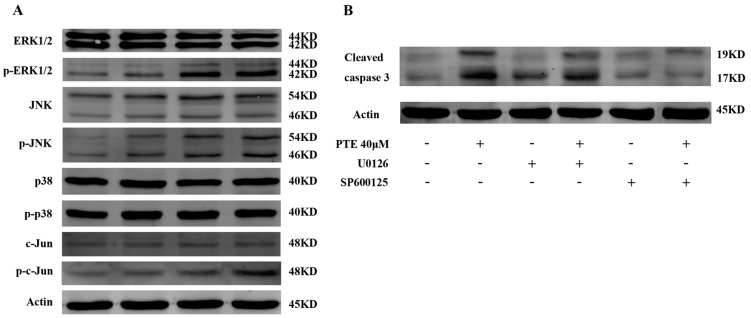
Effect of PTE on mitogen-activated protein kinase (MAPK) signaling pathway. (**A**) H929 cells were treated with PTE (10, 20, and 40 μM) for 48 h and the levels of extracellular regulated protein kinases (ERK) 1/2, phospho-ERK1/2, p38 MAPK, phospho-p38 MAPK, c-Jun N-terminal kinase (JNK), phospho-JNK, c-Jun and phospho-c-Jun assessed by Western blot; and (**B**) pretreatment with U0126, and SP600125 (10 μM each) for 3 h, after which H929 cells were treated with 40 μM PTE and the levels of cleaved caspase 3 assessed by Western blot.

To further investigate the molecular mechanism by which PTE exerts an anti-tumor effect on MM cells, we analyzed several potential pathways by Western blot. PTE enhanced the activation of phosphorylated extracellular regulated protein kinases (ERK) 1/2 and c-Jun N-terminal kinase (JNK) (Figure 5A). Indeed, the expression of c-Jun, a downstream protein of JNK, was increased after treatment with PTE. However, the expression of phosphorylated p38, a kinase involved in the mitogen-activated protein kinase (MAPK) pathway remained unchanged. To further prove that ERK1/2 and JNK are involved in PTE-induced apoptosis, H929 cells were pretreated with U0126 (ERK1/2 inhibitor) or SP600125 (JNK inhibitor), and activation of cleaved caspase 3 is decreased, suggesting that ERK1/2 and JNK inhibitors can block the PTE-induced apoptosis (Figure 5B). 

### 2.7. The Anti-Tumor Effect of PTE In Vivo

**Figure 6:**
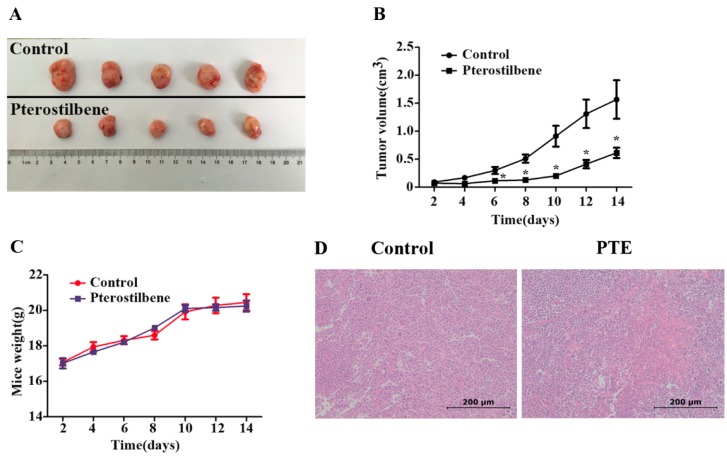
PTE inhibits the growth of implanted H929 cells in a xenograft mouse model. H929 cells (3 × 10^6^) were subcutaneously implanted into the flank of four-week-old female non-obese diabetes/severe combined immunodeficiency (NOD/SCID) mice and administered 5% dimethyl sulphoxide (DMSO) and saline or PTE (50 mg/kg) for 14 days (*n* = 5/group). (**A**) tumor samples were collected and imaged using a high-definition digital camera; (**B**) tumor volume was measured each day for 14 days (* *p* < 0.05); (**C**) mouse weight was measured for 14 days; and (**D**) haematoxylin and eosin (H & E) staining of tumors from DMSO or PTE treated mice (original magnification: 200×).

To further validate the anti-tumor effect of PTE, we injected H929 cells into the flanks of non-obese diabetes/severe combined immunodeficiency (NOD/SCID) mice and treated them with 5% dimethyl sulphoxide (DMSO) (Sigma, St. Louis, MO, USA) and saline or PTE for 14 days via intraperitoneal injection. Administration of PTE resulted in a significant decrease in tumor volume compared to 5% DMSO and saline treated animals (Figure 6A,B), indicating that PTE effects tumor growth in vivo. Daily analysis of mouse weight revealed no significant differences between the treatment groups (Figure 6C). Additionally, haematoxylin and eosin (H & E) staining demonstrated increased necrosis in the tumors of the PTE-treated group compared with the 5% DMSO and saline treated tumors (Figure 6D).

## 3. Discussion

PTE is a natural analog of resveratrol isolated from blueberries, which has a potent anti-tumor effect in cancer both in vitro and in vivo [12,15,16]. However, to date, the anti-tumor effect of PTE on MM cells is yet to be reported. In the present study, we demonstrate that the anti-tumor action of PTE on MM cell lines is associated with the induction of apoptosis and cell cycle arrest in vitro. In keeping with this, administration of PTE in vivo was found to inhibit tumor growth in tumor inoculated mice.

Apoptosis and cell cycle arrest can induce the inhibition of cell proliferation. Here, we assessed apoptosis in MM cell lines, primary CD138^+^ MM cells and normal PBMCs following PTE treatment and found that PTE induces apoptosis in MM cell lines and primary CD138^+^ MM cells. Furthermore, PTE increased the expression of cleaved caspase-3, cleaved caspase-8 and cleaved caspase-9, suggesting that PTE induced apoptosis of MM cells is caspase dependent and may be through the activation of both extrinsic and intrinsic apoptotic pathways. Caspase-3 is the most important component in the apoptotic pathway, and can be activated by caspase-8 or caspase-9, the two key proteins of the extrinsic and intrinsic apoptotic pathways, respectively [17]. In order to further investigate the relationship between PTE and the intrinsic apoptotic pathway, we assessed mitochondrial membrane potential (MMP). MMP is an indicator of mitochondrial membrane permeability, which is decreased during early apoptosis leading to activation of caspase-9 [18]. Additionally, mitochondria have been proposed as a new target for cancer intervention and treatment [19]. These results indicate that PTE can also induce cell apoptosis via the intrinsic apoptotic pathway as reflected by the PTE mediated loss of MMP. In addition, we demonstrated that PTE had no effect on normal PBMCs, supporting the potential of PTE as a safe and non-toxic natural compound.

It has been reported that PTE induces apoptosis by influencing ROS generation in malignant tumors [8,9,15]. ROS are comprised of the superoxide radical anion, hydrogen peroxide (H_2_O_2_), and the hydroxyl radical, with excessive ROS production associated with DNA damage and cell death by pyrimidine and purine bases and single-strand breaks [20]. In addition, ROS is reported to be an integral second messenger for cell signaling [21]. As mitochondria are particularly sensitive to ROS, we assessed changes in ROS generation following PTE treatment and found that PTE significantly increased ROS production. Furthermore, PTE treatment resulted in increased expression of the DNA damage marker, γ-H2AX, indicating that PTE increases DNA damage in H929 cells by ROS generation.

In agreement with recent research reporting the anti-tumor effect of PTE on various malignant cancers [22], we demonstrated that PTE can arrest cell cycle at G0/G1 phase. Additionally, protein analysis revealed that PTE increased the expression of p-CHK1, p-CHK2 and p21 and decreased the expression of CDK4, CDK6 and cyclin-D1. The cyclin-D1/CDK4/6 complex is the most important complex in the G0/G1 phase that regulates the progression from the G0/G1 phase to the G2/M phase [23]. When cyclin-D1/CDK4/6 complex is inhibited, the cell cycle is arrested in G0/G1 phase, leading to the inhibition of cell proliferation and promotion of apoptosis. CHK1 and CHK2, the G1/S checkpoints, are integral components of the cell cycle that regulate the process from G1 phase to S phase by enhancing the transcription of p21, a CDK inhibitor that can inhibit most cyclin/CDK complexes [24]. Conversely, CHK1 and CHK2 play an important role in the DNA damage response pathway, which is activated in response to DNA damage and rapidly induces cell cycle arrest and DNA damage repair mechanisms [25].

Several pathways are reported to be involved in PTE-induced apoptosis. Here, we demonstrate that PTE enhances the activation of ERK1/2 and JNK without any obvious effect on p38, a component of the MAPK pathway. This is in agreement with previous research describing the effect of PTE on human acute myeloid leukemia cell lines that PTE can induce the activation of ERK1/2 and JNK [14]. Analysis of the JNK/c-Jun pathway uncovered that PTE also induces activation of c-Jun. The MAPK pathway includes ERK1/2, p38 and JNK, and is known to regulate cell proliferation, differentiation, apoptosis and tumorigenesis in various types of cancers. Moreover, many chemotherapeutic agents induce an anti-tumor effect by regulating the MAPK pathway [26,27]. Taken together, these results indicate that activation of ERK1/2 and JNK plays an important role in PTE-induced apoptosis in MM cells.

It is well established that PTE has an anti-tumor effect in vitro in human melanoma [8], esophageal cancer [15] and prostate cancer [12], which is consistent with the anti-tumor effect observed in MM cell lines in the present study. Furthermore, PTE has been reported to decrease tumor size and prolong survival in tumor-inoculated mice [28,29,30]. In the present study, mice were administered 5% DMSO and saline or 50 mg/kg PTE via intraperitoneal injection for 14 days, resulting in a significant reduction in tumor volume with PTE treatment. Additionally, no differences were observed in mouse weight between treatment groups, suggesting that PTE may be well tolerated in vivo. Taken together, the results of this study reveal that PTE is a potent anti-tumor agent in MM cells in vitro and in vivo, is associated with low toxicity and thus may represent potential novel therapy for treating MM patients.

## 4. Materials and Methods

### 4.1. Cell Culture

H929 [31] and RPMI-8226 [32] were purchased from the American type culture collection (ATCC) (Manassas, VA, USA). ARP-1 [33] and OCI-MY5 [34] were a kind gift from Fenghuang Zhan (Department of Internal Medicine, University of Iowa, Iowa City, IA, USA). Cells were cultured in RPMI-1640 (Gibco, Carlsbad, CA, USA), supplemented with 10% fetal bovine serum (Gibco) and 1% penicillin-streptomycin (Gibco) and maintained at 37 °C in 5% CO_2_. Culture medium was changed every other day. Primary CD138^+^ MM cells were obtained from the bone marrow of three initial diagnosis MM patients via using magnetic bead selection (Miltenyi Biotech, Auburn, CA, USA) after being isolated with lymphoprep (Stemcell Technologies, Vancouver, BC, Canada). Normal PBMCs were isolated from the human peripheral blood using lymphoprep. Primary CD138^+^ MM cells and normal PBMCs were maintained in RPMI 1640 medium containing 10% FBS. Informed consent was obtained from each patient. These studies have been approved by the institutional review board of Shanghai Tenth People’s Hospital, Shanghai, China.

### 4.2. Cell Proliferation Assay

MM cells H929, RPMI-8226, ARP-1 and OCI-MY5 were seeded at a density of 2 × 10^4^ cells per well and treated with 10, 20, 30, 40 or 50 μM of PTE in a 96-well plate for 24, 48 and 72 h. Prior to reading, 10 μL of cell counting kit (CCK)-8 (Yeasen, Shanghai, China) was added to each well and incubated at 37 °C for 2 h. Cells were then measured on a microplate reader (Synergy H4, BioTek, Winooski, VT, USA) at 450 nm to assess proliferation. The half maximal inhibitory concentrations (IC_50_) of the MM cells in response to treatment were calculated using CalcuSyn software (Beijing HuanZhongRuiChi Technology Co., Beijing, China).

### 4.3. Cell Apoptosis Assay

H929 cells were seeded at a density of 2 × 10^5^ cells per well in a 24-well plate and treated with 0, 10, 20 or 40 μM of PTE for 24, 48 or 72 h. Primary CD138^+^ MM cells and normal PBMCs were treated with PTE for 48 h. Cells were subsequently washed with PBS and stained with the Annexin V-FITC Apoptosis Detection Kit (BD BioScience, San Jose, CA, USA) protected from light, and analyzed by a BD FASCCanto II flow cytometer (BD). *N*-acetyl-l-cysteine (NAC) was from Beyotime (Shanghai, China). The pan-caspase inhibitor Z-VAD-FMK was from Selleckchem (Houston, TX, USA). 

### 4.4. Cell Cycle Assay

H929 cells were cultured in serum-free medium for 12 h at a density of 2 × 10^5^ cells per well, and then placed in a complete medium and treated with 0, 10, 20 and 40 μM of PTE for 6 h prior to fixation in cold 75% alcohol overnight. The cells were stained with propidium iodide (PI) (BD) for 15 min at room temperature (RT), washed with PBS and analyzed on the flow cytometer.

### 4.5. Measurement of ROS Generation

H929 cells were treated with 10 μM of PTE for 24 h at 37 °C, washed in PBS, resuspended in RPMI-1640 medium containing 10 μM of 2′,7′-dichlorofluorescin diacetate (DCFH-DA) (Sigma, St. Louis, MO, USA), and incubated at 37 °C for 30 min. Fluorescence intensity was measured using a flow cytometer.

### 4.6. JC-1 Assay

H929 cells were treated with 40 μM of PTE for 24 h at 37 °C and stained with 10 μM of JC-1 according to the manufacturer instructions (Life Technologies, Carlsbad, CA, USA). The cells were analyzed using a flow cytometer. Mitochondrial membrane potential results were expressed as the percentage of cells that were double positive. CCCP (carbonyl cyanide 3-chlorophenylhydrazone, supplied with the kit) controls were used to confirm that the JC-1 response was sensitive to changes in membrane potential.

### 4.7. Western Blot Analysis

Cells were lysed in radio-immunoprecipitation assay (RIPA) buffer (Sigma) and proteins were migrated on 10% or 12.5% SDS-PAGE gels and transferred to nitrocellulose or polyvinyldifluoride membranes. The membranes were blocked in 5% low fat milk for 1 h, and probed with the relevant primary antibodies overnight at 4 °C. The primary antibodies γ-H2AX, cleaved caspase-3, cleaved caspase-8, caspase-9, phospho-CHK1, phospho-CHK2, cyclin-D1, CDK6, CDK4, p21, ERK1/2, phospho-ERK1/2, p38 MAPK, phospho-p38 MAPK, JNK, phosphor-JNK, c-Jun, phospho-c-Jun and β-actin were obtained from Cell Signaling Technology (CST) (Beverly, MA, USA). Membranes were then probed with the appropriate secondary antibodies (CST) for 1 h at RT. The membranes were revealed using the Odyssey two-color infrared laser imaging system (LICOR, Lincoln, NE, USA). The ERK1/2 inhibitor U0126 and JNK inhibitor SP600125 were from Selleckchem.

### 4.8. Tumor Xenograft Models

Female NOD/SCID mice (4 weeks old) were purchased from Shanghai Laboratory Animal Center (Shanghai, China). Mice were housed in a standard animal laboratory and fed a standard diet with free access to water. H929 human MM cells (3 × 10^6^) in 100 μL of serum-free culture medium were subcutaneously injected into the upper flank region of the NOD/SCID mice. On the eighth day, 10 mice were randomly divided into the control (5% DMSO and saline) or PTE group (50 mg/kg PTE in 5% DMSO and saline) (*n* = 5/group). Mice were injected with 5% DMSO and saline or PTE for 14 days and assessed for tumor size and weight each day. At the end of the experiment, mice were euthanized and the tumors were imaged. Tumor volume was calculated using the following formula: V = A × B^2^/2 (A = largest diameter; B = smallest diameter). Following sacrifice, tumors were removed from each mouse, fixed in 4% paraformaldehyde (PFA) for 24 h and embedded in paraffin; in addition, 5 μm-thick sections were prepared and stained with H & E. All animal studies have been approved by the institutional review board of Shanghai Tenth People’s Hospital (ID:SYXK 2011-0111).

### 4.9. Pterostilbene

PTE was purchased from J & K Chemical Ltd. (Beijing, China) with purity of 98%. The compound was dissolved in DMSO to yield a 100 mM stock solution and stored at −20 °C. The different doses of PTE (10, 20, 30, 40, 50 μM) in treatment groups were diluted with RPMI-1640, while to the control group was added the corresponding volume of DMSO and RMPI-1640.

### 4.10. Statistical Analysis

Data are expressed as mean ± standard deviation (SD). Student’s *t*-test and one-way analysis of variance (ANOVA) were performed as appropriate using SPSS v20.0 software (IBM, Armonk, NY, USA). A *p*-value of *p* < 0.05 was considered statistically significant.

## 5. Conclusions

Taken together, the results of our study demonstrate that PTE exerts an anti-tumor effect on MM cell lines both in vitro and in vivo. PTE inhibited cell proliferation, enhanced apoptosis and caused cell cycle arrest through ROS generation and DNA damage, and was associated with activation of ERK1/2 and JNK signaling. Given that MM has a poor prognosis and is associated with resistance to therapy, novel compounds that effectively target MM are of great interest in order to improve patient outcomes. As PTE is a natural compound that exhibits low toxicity, PTE represents a promising potential treatment for MM patients.
